# The efficacy of various forms of premedication in dental procedures for patients with schizophrenia

**DOI:** 10.1192/j.eurpsy.2026.12193

**Published:** 2026-07-31

**Authors:** M. S. Artemieva, V. Guts, D. Shumeyko

**Affiliations:** 1 Peoples’ Friendship University of Russia named after Patrice Lumumba, Moscow, Russian Federation; 2 Salius medical group, Prague, Czech Republic

## Abstract

**Introduction:**

Patients with schizophrenia are particularly susceptible to stress, including dental trauma, under the influence of which there is a risk of both exacerbation of mental illness and the occurrence of adverse reactions caused by dental procedures, including refusal of treatment. According to literary sources, the issues of premedication in mentally ill patients before dental treatment have not been sufficiently developed.

**Objectives:**

In this regard, the aim of the work was to develop methods for assessing the effectiveness of psychotherapeutic and psychopharmacological preparation of patients with schizophrenia for dental procedures, the choice of methods of psychocorrection and premedication in accordance with the mental status of the patient.

**Methods:**

The work used clinical-psychopathological, clinical-anamnestic methods. For express assessment of psychoemotional state of patients with schizophrenia before dental procedures before and after premedication, the Clinical Dental Scale (CDS, 1983, A.F. Bizyaev, S.Yu. Ivanov) was used.

**Results:**

Based on the conducted studies, it was found that without a psychoprophylactic conversation, only 5% of patients with schizophrenia underwent adequate dental procedures; after a psychotherapeutic conversation, instances of successful interventions increased 3 times. For premedication, in accordance with the instructions of the attending psychiatrist, drugs from the group of tranquilizers or neuroleptics were prescribed.

**Conclusions:**

The conducted studies indicate that the method of express assessment of the psychoemotional state of patients with schizophrenia before dental procedures allows identifying patients with moderate and severe psychoemotional stress and choosing the most effective premedication method.

**Disclosure of Interest:**

None Declared

